# Hepatocellular Carcinoma in the Pediatric Population: A Population Based Clinical Outcomes Study Involving 257 Patients from the Surveillance, Epidemiology, and End Result (SEER) Database (1973–2011)

**DOI:** 10.1155/2015/670728

**Published:** 2015-11-18

**Authors:** Christine S. M. Lau, Krishnaraj Mahendraraj, Ronald S. Chamberlain

**Affiliations:** ^1^Department of Surgery, Saint Barnabas Medical Center, Livingston, NJ 07039, USA; ^2^Saint George's University School of Medicine, True Blue, Grenada; ^3^Department of Surgery, New Jersey Medical School, Rutgers University, Newark, NJ 07103, USA

## Abstract

*Introduction*. Hepatocellular carcinoma (HCC) is a rare pediatric cancer accounting for 0.5% of all pediatric malignancies. This study examines a large cohort of HCC patients in an effort to define the factors impacting clinical outcomes in pediatric HCC patients compared to adults.* Methods*. Demographic and clinical data on 63,771 HCC patients (257 pediatric patients ≤ 19 and 63,514 adult patients age ≥ 20) were abstracted from the SEER database (1973–2011).* Results*. HCC was more common among males (59.5% pediatric and 75.1% adults) and Caucasians (50.4% and 50.5%), *p* < 0.05. Children more often presented with fibrolamellar variant HCC (24.1% versus 0.3%, *p* = 0.71) and advanced HCC, including distant disease (33.1% versus 20.8%, *p* < 0.001), and tumors > 4 cm in size (79.6% versus 62.0%, *p* = 0.02). Pediatric HCC patients undergoing surgery (13.107 versus 8.324 years, *p* < 0.001) had longer survival than adult HCC patients. Overall mortality was lower (65.8% versus 82.0%, *p* < 0.001) in the pediatric HCC group.* Conclusion*. HCC is a rare pediatric malignancy that presents most often as an advanced tumor, >4 cm in Caucasian males. Children with HCC achieve significantly longer mean overall survival compared to adults with HCC, primarily attributable to the more favorable fibrolamellar histologic variant, and more aggressive surgical intervention, which significantly improves survival.

## 1. Introduction

Primary liver neoplasms in childhood are rare and constitute 1-2% of all pediatric tumors [1]. Hepatocellular carcinoma (HCC) is the second most common primary hepatic malignancy in children following hepatoblastoma and accounts for approximately one-third of all primary hepatic malignancies in children and approximately 0.5% of all pediatric malignancies [2]. The age-standardized rates of HCC range from approximately 7.5 per 100,000 men in North America to as high as 20 per 100,000 men in Eastern and Southeastern Asia [3, 4]. HCC in children is much rarer, with an age-adjusted incidence rate of 0.7 per 1,000,000 in the USA [5]. The incidence of HCC is significantly greater in Eastern and Southeastern Asia and in Africa, where hepatitis B infection is endemic [3]. HCC has also been observed in young children with inherited metabolic disorders and in older children with maternal transmission of hepatitis B infection [5–8].

While surgery remains the mainstream of treatment for patients with HCC, survival rates are poor [9]. In a series of 218 pediatric HCC cases, Allan et al. reported an overall 5-year survival of 24% and a 20-year survival of only 8% [10].

HCC in the pediatric population is much less prevalent than in adults. Current knowledge regarding pediatric HCC is limited, and very few studies have examined treatment approaches and outcomes in children with HCC. This study sought to examine a large cohort of adult and pediatric HCC from the Surveillance, Epidemiology, and End Results (SEER) database, in an effort to identify demographic, clinical, and treatment strategies which impact clinical outcomes and potentially guide therapeutic decision-making and assist in clinical trial development and appropriate accrual.

## 2. Methods

Data for the current study was extracted from the Surveillance, Epidemiology, and End Result (SEER) database provided by the National Cancer Institute between 1973 and 2011. SEER Stat software version 8.0.4 was utilized to extract data from 18 SEER registries (Alaska Native Tumor Registry, Arizona Indians, Cherokee Nation, Connecticut, Detroit, Georgia Center for Cancer Statistics, Greater Bay Area Cancer Registry, Greater California, Hawaii, Iowa, Kentucky, Los Angeles, Louisiana, New Jersey, New Mexico, Seattle-Puget Sound, and Utah).

63,771 patients with histologically confirmed hepatocellular carcinoma were identified and exported to IBM SPSS v20.2. Patients with a primary diagnosis of HCC were identified to form the final study cohort, using the SEER International Classification of Disease for Oncology (ICD-O-3) codes 8170/3, 8171/3, 8172/3, 8173/3, 8174/3, and 8175/3. Demographic and clinical data extracted included age, gender, race, tumor morphology, stage, grade, size, and type of treatment received (surgery, radiation, both or no treatment). The 63,771 patients included in this study were grouped into pediatric patients (defined as age ≤ 19 years) and adult patients (defined as age ≥ 20 years). Patients with* in situ* cancers, those with nonspecific site of tumor origin, and those in whom histologic confirmation of their cancer was not available were excluded from the final study cohort. Endpoints examined included 1-, 2-, and 5-year overall survival, mortality, and cancer-specific mortality. Categorical variables were compared using the Chi-Square test, and continuous variables were compared using Student's *t*-test and analysis of variance (ANOVA). Multivariate analysis using the “backward Wald” method was performed to calculate odds ratios (OR) and determine independent factors affecting survival. Missing and unknown data were excluded from the multivariate analysis. Kaplan-Meier analysis was used to compare long term actuarial survival between groups. Statistical significance was accepted at the level of *p* < 0.05.

## 3. Results

### 3.1. Demographic Data

63,771 cases of HCC were reported in the SEER database between 1973 and 2011. The mean age at diagnosis was 64 ± 13 years. There were significantly more adult patients than pediatric patients (99.6% versus 0.4%). 99.6% of cases (*N* = 63, 514) occurred in adults with a mean age at diagnosis of 64 ± 12 years, while only 0.4% of cases (*N* = 257) occurred in pediatric patients with a mean age at diagnosis of 13 ± 5 years (Table 1).

The majority of HCC diagnoses occurred in Caucasians (*N* = 32, 130; 50.5%), followed by Asian, Pacific Islander or Native Americans (*N* = 13, 347; 21.0%), Hispanics (*N* = 10, 285; 16.2%), and African Americans (*N* = 7, 829; 12.3%), *p* < 0.05. HCC was more common among Caucasians in both the pediatric and adult population groups (50.4% and 50.5%, *p* = 0.03); however, pediatric HCC was significantly more common among Hispanics (19.3% versus 16.2%, *p* = 0.03) and less common among African Americans (11.4% versus 12.3%, *p* = 0.02) and Asian/Pacific Islander/Native Americans (18.9% versus 21.0%, *p* = 0.03), compared to adult HCC.

Among all 63,771 HCC patients, 75.1% were male (*N* = 47, 862), and 24.9% were female (*N* = 15, 909), resulting in a male to female ratio of 3.01 : 1, *p* < 0.001. HCC was more common among males in both the pediatric and adult populations (59.5% and 75.1%). Among the 257 pediatric HCC patients, 59.5% were males (*N* = 153) and 40.5% were females (*N* = 104), resulting in a male to female ratio of 1.47 : 1, *p* < 0.001. Among the 63,514 adult HCC patients, 75.1% were males (*N* = 47, 709) and 24.9% were females (*N* = 15, 805), resulting in a male to female ratio of 3.02 : 1, *p* < 0.001.

### 3.2. Tumor Characteristics

Among pediatric patients, 24.1% (*N* = 62) had fibrolamellar HCC (fHCC), 1.2% (*N* = 3) had clear cell HCC, and the remaining 74.7% (*N* = 192) had HCC NOS (Table 2). Morphology was more varied among adult HCC patients. 0.5% (*N* = 336) had clear cell HCC, 0.3% (*N* = 212) had fHCC, 0.1% (*N* = 66) had scirrhous HCC, 0.1% (*N* = 36) had spindle cell HCC, <0.1% (*N* = 17) had pleomorphic HCC, and 98.9% (*N* = 62, 847) had HCC NOS. The fHCC variant was far more common among pediatric patients, compared to adult patients (24.1% versus 0.3%, *p* = 0.71).

47.9% (*N* = 25, 755) of HCC cases presented with localized disease, 31.2% (*N* = 16, 777) had regional involvement, and 20.8% (*N* = 11, 193) had metastatic spread at the time of diagnosis, *p* < 0.001. Pediatric patients had a higher rate of regional disease (38.8% versus 31.2%, *p* < 0.001) and distant disease (33.1% versus 20.8%, *p* < 0.001) and a lower rate of localized disease (28.1% versus 48.0%, *p* < 0.001), compared to adult patients.

62.0% (*N* = 24, 255) of HCC were >4 cm, 30.0% (*N* = 11, 720) were 2–4 cm, 7.9% (*N* = 3, 071) were <2 cm, and 0.1% (*N* = 45) were microscopic lesions, *p* < 0.05. A greater percentage of pediatric patients had a tumor size greater than 4 cm (79.6% versus 62.0%, *p* = 0.02) compared to adult patients.

Overall, 38.0% (*N* = 8, 467) of HCC were moderately differentiated, 35.1% (*N* = 7, 813) were well differentiated, and 23.9% (*N* = 5, 317) were poorly differentiated, *p* < 0.05. 3.0% (*N* = 672) were undifferentiated, *p* = 0.06. Pediatric patients more often presented with well differentiated tumors (35.8% versus 35.1%, *p* = 0.02) and less often with moderately (34.7% versus 38.0%, *p* = 0.01) or poorly differentiated tumors (23.2% versus 23.9%, *p* = 0.01).

### 3.3. Treatment

20.9% (*N* = 12, 769) of all HCC patients were treated with surgery alone while 4.0% (*N* = 2, 459) of patients received radiation alone, and 0.5% (*N* = 330) of patients were treated with both surgery and radiation. 74.5% (*N* = 45, 558) of patients received no treatment, *p* < 0.005 (Table 3). Pediatric patients were more likely to receive treatment (52.6% versus 25.3%, *p* < 0.005) compared to adult patients. Among those receiving treatment, surgical resection alone was the most common treatment modality in both pediatric and adult patients (92.4% and 82.0%). More pediatric patients received surgery as primary treatment (48.6% versus 20.8%, *p* < 0.001).

Patients who received surgery as the primary modality of treatment experienced significant survival benefit (mean survival 8.560 ± 0.297 years), compared to those who received a combination of surgery and radiation (3.649 ± 0.499 years), primary radiation only (1.190 ± 0.064 years), or no treatment (1.253 ± 0.046 years), *p* < 0.005. Pediatric patients who received surgery (13.107 ± 1.306 years versus 8.324 ± 0.302 years, *p* < 0.001) or a combination of both surgery and radiation (13.667 ± 13.500 years versus 3.287 ± 0.387 years, *p* < 0.005) had significantly longer survivals than adult patients. Pediatric HCC patients who received combination surgery and radiation had similar survival compared to surgery alone (13.667 ± 13.500 years versus 13.107 ± 1.306 years). In contrast, adult HCC patients who received combination surgery and radiation had worse survival than those receiving surgery alone (3.287 ± 0.387 years versus 8.324 ± 0.302 years). Survival was slightly better among pediatric patients receiving radiation only (1.807 ± 0.447 years versus 1.189 ± 0.064 years, *p* < 0.001) or no treatment (2.072 ± 0.598 years versus 1.243 ± 0.046 years, *p* < 0.005), compared to their adult counterparts.

Overall, the fHCC variant was associated with the greatest survival (6.905 ± 0.617 years). Pediatric fHCC experienced significant longer survival than adult fHCC (9.110 ± 1.204 years versus 6.016 ± 0.653 years, *p* = 0.002).

### 3.4. Outcomes

**Figure 1:**
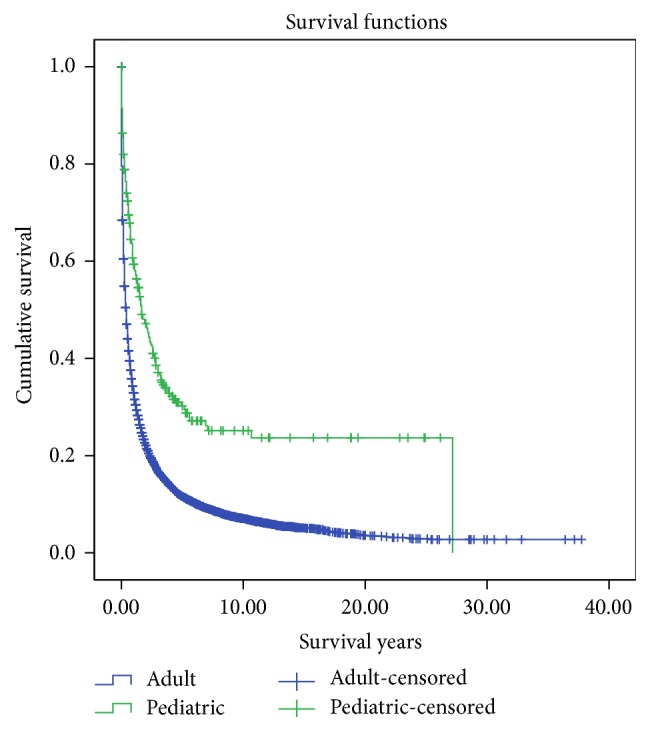
Kaplan-Meier curves illustrating actuarial survival for pediatric and adult patients with hepatocellular carcinoma from the Surveillance, Epidemiology, and End Results (SEER) database, 1973–2011.

The mean actuarial survival for pediatric patients was significantly higher than adult patients (7.988 ± 0.845 years versus 2.781 ± 0.075 years, *p* < 0.001). For all HCC patients, the overall and cancer specific mortality were found to be 81.9% and 62.7%, respectively, *p* < 0.001 (Table 3). Overall and cancer-specific mortality among the pediatric patients (65.8% and 60.7%) were significantly lower compared with adult patients (82.0% and 62.7%), *p* < 0.001. Cumulative 1-, 2- and 5-year survival rates were significantly higher amongst the pediatric patients (61%, 47%, and 30%) as compared to adult patients (34%, 23%, and 12%), *p* < 0.001. Kaplan-Meier curve illustrates significantly better 40-year actuarial survival for pediatric HCC patients compared to adults (Figure 1).

### 3.5. Fibrolamellar versus Nonfibrolamellar Hepatocellular Carcinoma

274 cases of fHCC were reported, with a mean age of 64 ± 12 years, representing 0.4% of all HCC cases. 22.6% of fHCC cases (*N* = 62) were pediatric patients, while 77.4% (*N* = 212) were adults. 24.1% of pediatric patients had fHCC, compared to 0.3% of adults. fHCC was more common among males in both pediatric and adult populations with a male to female ratio of 1.21 : 1 among pediatric patients and 1.59 : 1 among adults (Table 4). Non-fHCC had a much higher prevalence among males, with male-to-female ratios of 1.57 : 1 among pediatric patients and 3.03 : 1 among adults, *p* < 0.001.

37.2% (*N* = 96) of fHCC cases presented as localized disease, 33.3% (*N* = 86) had regional disease, and 29.5% (*N* = 76) had distant disease (Table 5). fHCC had a higher rate of regional disease (33.3% versus 31.2%, *p* < 0.001) and distant disease (29.5% versus 20.8%, *p* < 0.001) and a lower rate of localized disease (37.2% versus 48.0%, *p* < 0.001), compared to non-fHCC. Most fHCC were moderately differentiated tumors (50.0%; *N* = 41), while 26.8% (*N* = 22) were well differentiated, and 23.2% (*N* = 19) were poorly differentiated. fHCC had higher rates of moderately differentiated tumors (50.0% versus 38.0%, *p* > 0.05) and similar rates of poorly differentiated tumors (23.2% versus 23.9%, *p* > 0.05), but lower rates of well differentiated tumors (26.8% versus 35.1%, *p* = NS). A greater proportion of fHCC had tumors > 4 cm (86.8% versus 61.9%, *p* > 0.05). 90.9% of pediatric fHCC presented with tumors > 4 cm, while 85.3% of adult fHCC presented with tumors > 4 cm.

A greater proportion of fHCC patients received treatment (54.9% versus 25.3%, *p* < 0.001), compared to non-fHCC (Table 6). Surgery was the most common treatment modality utilized in both fHCC and non-fHCC; however, significantly more fHCC patients received surgery alone (49.6% versus 20.8%) or combination of surgery and radiation (2.6% versus 0.5%, *p* < 0.001), compared to non-fHCC. A greater proportion of fHCC received surgical resection alone in both pediatric (66.1% versus 42.8%, *p* < 0.001) and adult populations (44.7% versus 20.7%, *p* < 0.001), compared to non-fHCC.

Patients with fHCC treated with surgery (mean survival 11.325 ± 0.966 years) and combination of surgery and radiation (7.393 ± 2.937 years) experienced significant survival benefit, compared to those who received radiation only (1.111 ± 0.348 years) or no treatment (1.916 ± 0.464 years), *p* < 0.001 (Table 7). Patients with fHCC treated with surgery (11.325 ± 0.966 years versus 8.345 ± 0.296 years) and combination surgery and radiation (7.393 ± 2.937 years versus 3.475 ± 0.489 years) had longer survivals than patients with non-fHCC, *p* < 0.001.

**Figure 2:**
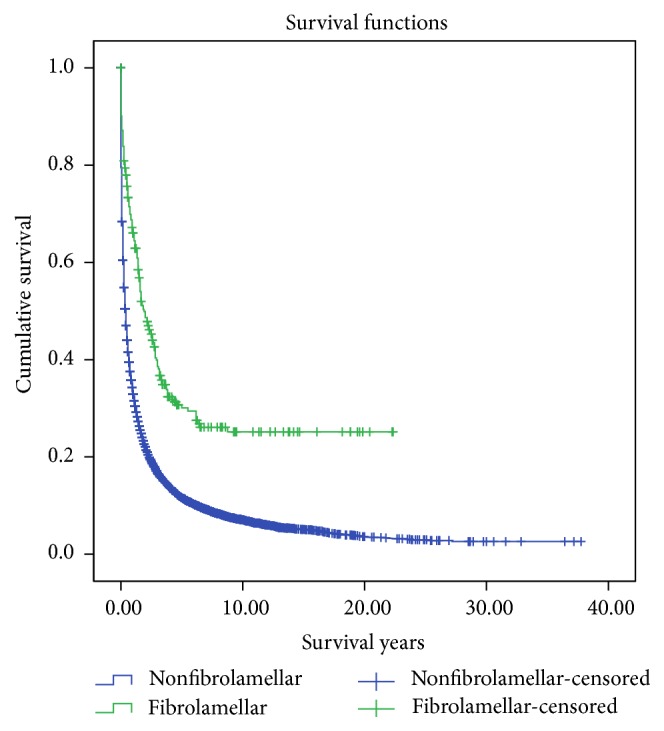
Kaplan-Meier curves illustrating actuarial survival for patients with fibrolamellar and nonfibrolamellar hepatocellular carcinoma from the Surveillance, Epidemiology, and End Results (SEER) database, 1973–2011.

Overall (66.1% versus 82.0%, *p* < 0.001) and cancer-specific mortality (56.2% versus 62.8%, *p* < 0.001) were lower among fHCC patients, compared to non-fHCC. fHCC had a higher mean overall survival in both pediatric patients (9.110 ± 1.1204 years versus 6.424 ± 0.866 years) and adult patients (6.016 ± 0.653 years versus 2.657 ± 0.072 years), compared to non-fHCC. Patients with fHCC also had longer 1-year, 2-year, and 5-year cumulative survival (67%, 50%, 31%) compared to non-fHCC (34%, 23%, 12%). Kaplan-Meier curve illustrates significantly better 20-year actuarial survival for fHCC compared to non-fHCC (Figure 2).

### 3.6. Multivariate Analysis

Multivariate analysis identified regional disease (OR 1.9, CI = 1.1–3.1), distant disease (OR 4.5, CI = 2.3–8.8), and tumor size > 4 cm (OR 4.5, CI = 1.7–9.8) as being independently associated with increased mortality in the pediatric population, *p* < 0.005. Similarly, adults with distant disease (OR 6.5, CI = 4.3–9.1), poorly or undifferentiated disease (OR 7.5, CI = 5.3–9.6), and tumor size > 4 cm (OR 8.1, CI = 5.3–11.6) were found to have the highest odds of mortality on multivariate analysis, *p* < 0.005. fHCC was not found to be favorable in either pediatric, adult, or overall populations, *p* > 0.05. For the pediatric population with fHCC disease, distant disease (OR 3.2, CI = 2.2–5.7) and tumor size > 4 cm (OR 1.6, CI = 1.0–2.2) were independently associated with increased mortality, *p* < 0.001.

## 4. Discussion

The development of HCC is associated with multiple etiologies, high incidence rates, and high mortality [2]. The overall incidence of HCC varies from approximately 10 per 100,000 in North America to as high as 20 per 100,000 in countries where hepatitis B is endemic, such as Southeastern Asia [3, 4]. Based on previous studies, the incidence of pediatric HCC in the United States has remained relatively stable, with some authors documenting reduction in HCC rates of >10% following immunization against hepatitis B in endemic countries [10–13]. While adult HCC is often associated with chronic hepatitis B infection and liver cirrhosis, most pediatric HCC are* de novo* tumors and are not related to cirrhosis [14, 15]. In cases where pediatric HCC is associated with cirrhosis, it is most often in the context of biliary atresia, Fanconi's syndrome, and hepatitis B [6, 7, 14]. Pediatric HCC is also associated with metabolic diseases such as hereditary tyrosinemia and glycogen storage disease type IA [6, 7, 14].

Despite being the second most common primary pediatric liver malignancy following hepatoblastoma, HCC is rare in children and adolescents under the age of 20 and accounts for less than 1% of all HCC cases [14]. Similar to previously published data, this study identified a higher male HCC incidence rate for both pediatric and adult populations (59.5% and 75.1%) as well as finding that HCC was more prevalent among Caucasians in both populations [2, 5, 10, 15]. Interestingly, a higher percentage of pediatric HCC occurred among Hispanic patients, while much lower percentage of African Americans were affected. These results are consistent with previous retrospective studies conducted by McAteer et al. and Allan et al., in which over 60% of pediatric HCC occurred in Caucasians [2, 10]. These results suggest that Caucasian males in the pediatric population are at highest risk for HCC, and pediatricians should maintain a higher degree of suspicion for HCC when these patients present with suspicious or nonspecific signs and symptoms.

This study identified that pediatric patients present with more advanced disease and larger tumor sizes compared to adult HCC patients. Pediatric patients also exhibited higher rates of regional and distant disease, and lower rates of localized disease compared to adults. A greater percentage of pediatric patients had tumors larger than 4 cm compared to adults. These results are similar to the findings of Allan et al., which reported that 35% of pediatric patients had regional disease, 34% had distant disease, and only 27% had local disease [10]. The remaining 4% were unstaged [10]. Conversely, McAteer et al. reported that most children affected with HCC presented with localized and regional disease (45.6% and 35.4% resp.), compared to distant disease (19.0%) [2]. This disparity can be partly explained by the difficulty in diagnosing HCC in children. Most patients are asymptomatic until the tumor grows sufficiently large enough to cause abdominal symptoms or a palpable abdominal mass [1, 14, 16, 17]. The vague symptoms associated with HCC usually do not result in limitations in daily activities or signs of hepatic insufficiency, contributing to a delay in diagnosis and likely accounting for its more advanced stages and larger size [16]. This study identified that fHCC was more likely to present with larger tumors as well as regional and distant disease. fHCC is the most common histology among pediatric patients, which could also account for the larger and less favorable stage distribution among pediatric HCC. Eggert et al. have previously reported on 47,040 HCC patients, including 183 fHCC patients, and also reported on greater proportions of fHCC presented with tumors > 5 cm (63% versus 34%) and distant disease (33% versus 13%), compared to non-fHCC, *p* < 0.0001 [18].

Surgical resection is the gold standard therapy for HCC [2, 14]. In this study, pediatric patients were far more likely to be treated with surgical resection than adults, even though surgical resection was associated with a significantly improved survival in all patients. While complete excision of the HCC tumor remains the only chance for cure and prolonged survival, rates of complete excision remain low [16]. Improved survival and high resectability rates in pediatric patients are at least in part due to a higher prevalence of the indolent fHCC variant among younger patients [18]. The significantly larger percentage of pediatric patients with fHCC observed in this study is consistent with a prior SEER study in which fHCC variant was the most common variant found in the pediatric population and accounted for 41.3% of surgically treated pediatric HCC and 25.4% of nonsurgically treated pediatric HCC [2]. In a retrospective SEER study by Allan et al. involving 218 patients (55 fHCC and 160 non-fHCC), fHCC was more likely to be treated with surgery (69% versus 46%, *p* = 0.003) and was associated with significantly improved survival, compared to non-fHCC [10]. Surgically resected fHCC was associated with significantly higher 10-year survival rates compared to non-fHCC (59% versus 37%, *p* = 0.002) [10].

Nonsurgical and adjuvant treatments for HCC, including chemoembolization, ethanol injection, and radiofrequency ablation, are often utilized when surgery is not possible or as a bridge to surgery with the goal of tumor shrinkage or for palliation in unresectable cancer [14]. Radiation treatment alone is rarely used for the treatment of HCC, and only 4% of patients in this study received radiation alone. Chemotherapy for HCC is limited, and HCC remains one of the most chemoresistant tumors [15]. Czauderna et al. studied 42 patients and reported that while 49% of patients obtained a partial response to chemotherapy, survival improved by less than 5% among responders [15]. These authors also reported a 5-year survival of 28% and event-free-survival (EFS) of 17% [15]. Zhang et al. studied 45 pediatric HCC patients and reported a median survival of 6 months [19]. Patients who had surgical resection had a significantly longer survival (median survival of 28.6 months) compared to those who received chemotherapy (4 months) or no treatment (5 months), *p* < 0.001 [19].

Although surgery is the primary therapy for all HCC, approximately 80% of HCC patients present with unresectable tumors, either due to large tumor burden, underlying hepatic dysfunction or metastatic disease [20]. Surgery is most beneficial in patients with good hepatic function and the absence of cirrhosis but is typically contraindicated in patients with extensive cirrhosis for when transplant may play a more critical role [20, 21]. Multiple studies have demonstrated that liver transplant confers both a significant survival advantage and a lower risk of recurrence versus surgical resection alone [22–24]. Very limited data is available on liver transplant for pediatric HCC; however, it also appears to improve overall survival in comparison to surgical resection [25–29]. Austin et al. studied 196 patients (41 patients with HCC and 135 patients with hepatoblastoma) from the United Network for Organ Sharing (UNOS) database and reported that the overall actuarial 1-, 5-, and 10-year survival for pediatric HCC liver recipients < 18 years of age were 86%, 63%, and 58%, which were similar to survival rates for pediatric liver transplants for hepatoblastoma (79%, 69%, 66%), *p* = 0.73 [25]. Metastatic or recurrent disease accounted for 86% of HCC deaths [25]. Despite the successes shown with liver transplantation, its use is substantially limited by organ availability.

Although HCC remain an aggressive malignancy typically associated with poor overall and cancer-specific survival, affected pediatric patients fare far better than their adult counterparts, with significantly improved mean overall survival as well as 1-, 2-, and 5-year cumulative survival. This is particularly interesting since pediatric patients typically present with more advanced disease and larger tumors. Prolonged survival among pediatric patients is likely at least partly a reflection of a much higher prevalence of the fHCC variant as well as lower rates of cirrhosis in affected children.

As with nearly all cancers, advanced stage at presentation makes it difficult to achieve total surgical resection and is therefore associated with poor prognosis [10, 14]. In the current study, distant disease and tumor sizes > 4 cm were associated with increased mortality in both children and adults, which is consistent with prior reports [2, 10].

Despite presenting with larger tumors and more advanced disease, fHCC was associated with significantly prolonged survival compared to non-fHCC in both the pediatric and adult population. The slow progressive, indolent disease course of fHCC accounts for the difficulty in diagnosing fHCC until later stages, as well as the prolonged survival [18]. Furthermore, fHCC patients are more likely to receive initial treatment with radiofrequency ablation, surgical resection, or transplantation. Similar results have been published in prior studies [10, 18]. Eggert et al. studied 47,040 HCC patients, including 183 fHCC patients, and reported that fHCC was associated with higher 5-year survival compared to non-fHCC (33.6% versus 16.1%) [18]. Allan et al. studied 218 pediatric HCC patients and reported significantly greater overall survival for fHCC compared to non-fHCC as demonstrated by 5-year Kaplan Meier curves (*p* = 0.002) [10]. Conversely, the Childhood Liver Tumor Strategy Group (SIOPEL) reported on 62 HCC patients (24 with fHCC and 38 with non-fHCC) and reported no significant difference in EFS at 3-year follow-up (22% versus 28%, *p* = 0.30) or 3-year overall survival (42% versus 33%, *p* = 0.24) between fHCC and non-fHCC [30]. They also reported that 42% of patients with non-fHCC died within 1 year, whereas only 2% of fHCC patients died [30].

There are several limitations of this study which should be taken into account. First, the SEER database did not accurately code for important clinical factors such as socioeconomic status, geography, tumor depth, method of diagnostic confirmation, hepatitis B vaccination status, liver transplantation information, and comorbidities such as hepatitis B infection and liver cirrhosis, which may have had an influence on survival. Second, information on diagnostic imaging and follow-up was lacking. Data on surgical and radiation therapy utilized was available in the SEER database; however information on surgical resection margins and chemotherapy received was not, and this limits the study's ability to comment on the impact of adjuvant or neoadjuvant therapy. There may also be an element of selection bias in this data set, since SEER registries are more likely to sample from urban rather than rural areas. Despite these limitations, the SEER database contains data from 26% of the United States population, and these findings can be generalized to the overall population.

## 5. Conclusion

Despite being the second most common primary hepatic malignancy in children, HCC remains a rare disease among pediatric patients, accounting for approximately 0.5% of all new pediatric malignancies, with a world-wide age-adjusted incidence of approximately 2 per 100,000 persons. Similar to adults, HCC occurs most commonly among Caucasian male children; however, more Hispanics and less African American children are affected. Given the rarity of pediatric HCC and the vague nonspecific presentation, diagnosing pediatric HCC is often delayed, resulting in more advanced disease and larger tumors. Despite this fact, a significantly greater proportion of pediatric patients are affected with the indolent fHCC histologic variant, which is more amenable to surgery. Surgery is the preferred treatment and significantly prolongs survival in affected patients. Although radiation is associated with poorer outcomes in the adult population, adjuvant radiation may prolong survival in the pediatric population. Given the limited number of patients who received radiotherapy in the current study, as well as the grim prognosis of pediatric HCC and the high overall and cancer-specific mortality associated with this disease, additional studies investigating the role of radiation in pediatric HCC treatment are required to more precisely identify its role in the treatment of these patients.

## Figures and Tables

**Table 1:** Demographic profiles of 63,514 adults and 257 pediatric patients with hepatocellular carcinoma from the Surveillance, Epidemiology, and End Results (SEER) database, 1973–2011.

Variables	Overall	Pediatric patients	Adult patients	*p* value
*N* (%)	63,771	257 (0.4%)	63,514 (99.6%)	—
Age, (Mean ± SD)	64 ± 13	13 ± 5	64 ± 12	<0.001
Mean overall survival (years)	2.833 ± 0.076	7.988 ± 0.845	2.781 ± 0.075	<0.001
Gender, *N* (%)				
Male	47,862 (75.1%)	153 (59.5%)	47,709 (75.1%)	<0.001
Female	15,909 (24.9%)	104 (40.5%)	15,805 (24.9%)	<0.001
Race, *N* (%)^*∗∗*^				
Caucasian	32,130 (50.5%)	128 (50.4%)	32,002 (50.5%)	0.03
African American	7,829 (12.3%)	29 (11.4%)	7,800 (12.3%)	0.02
Hispanic	10,285 (16.2%)	49 (19.3%)	10,239 (16.2%)	0.03
Asian/Pacific Islander/Native Americans	13,347 (21.0%)	48 (18.9%)	13,299 (21.0%)	0.03

*N* = number; SD = standard deviation; ^*∗∗*^data presented for patients with available information only.

**Table 2:** Tumor characteristics of 63,514 adults and 257 pediatric patients with hepatocellular carcinoma from the Surveillance, Epidemiology, and End Results (SEER) database, 1973–2011.

Variables	Overall	Pediatric patients	Adult patients	*p* value
*N* (%)	63,771	257 (0.4%)	63,514 (99.6)	—
Morphology, *N* (%)				
Pleomorphic	17 (<0.0%)	0 (0.0%)	17 (<0.0%)	0.63
Clear cell	339 (0.5%)	3 (1.2%)	336 (0.5%)	0.16
Spindle cell	36 (0.1%)	0 (0.0%)	36 (0.1%)	0.81
Scirrhous	66 (0.1%)	0 (0.0%)	66 (0.1%)	0.77
Fibrolamellar	274 (0.4%)	62 (24.1%)	212 (0.3%)	0.71
NOS	63,039 (98.9%)	192 (74.7%)	62,847 (98.9%)	0.09
Grade, *N* (%)^*∗∗*^				
Well differentiated	7,813 (35.1%)	34 (35.8%)	7,779 (35.1%)	0.02
Moderately diff.	8,467 (38.0%)	33 (34.7%)	8,434 (38.0%)	0.01
Poorly diff.	5,317 (23.9%)	22 (23.2%)	5,295 (23.9%)	0.01
Undifferentiated	672 (3.0%)	6 (6.3%)	666 (3.0%)	0.06
Stage, *N* (%)^*∗∗*^				
Localized	25,755 (47.9%)	68 (28.1%)	25,687 (48.0%)	<0.001
Regional	16,777 (31.2%)	94 (38.8%)	16,683 (31.2%)	<0.001
Distant	11,193 (20.8%)	80 (33.1%)	11,113 (20.8%)	<0.001
Tumor size, *N* (%)^*∗∗*^				
Microscopic	45 (0.1%)	0 (0.0%)	45 (0.1%)	0.01
Under 2 cm	3,071 (7.9%)	16 (9.9%)	3,055 (7.8%)	0.02
2 to 4 cm	11,720 (30.0%)	17 (10.5%)	11,703 (30.1%)	0.02
Over 4 cm	24,255 (62.0%)	129 (79.6%)	24,126 (62.0%)	0.02

cm = centimeters; diff. = differentiated; *N* = number; NOS = not otherwise specified; SD = standard deviation; ^*∗∗*^data presented for patients with available information only.

**Table 3:** Treatment and survival outcomes of 63,514 adults and 257 pediatric patients with hepatocellular carcinoma from the Surveillance, Epidemiology, and End Results (SEER) database, 1973–2011.

Variables	Overall	Pediatric patients	Adult patients	*p* value
*N* (%)	63,771	257 (0.4%)	63,514 (99.6%)	—
Mean overall survival (years ± SD)	2.833 ± 0.076	7.988 ± 0.845	2.781 ± 0.075	<0.001
Treatment, *N* (%)^*∗∗*^				
No treatment	45,558 (74.5%)	118 (47.4%)	45,440 (74.7%)	<0.005
Surgery only	12,769 (20.9%)	121 (48.6%)	12,648 (20.8%)	<0.001
Radiation only	2,459 (4.0%)	8 (3.2%)	2,451 (4.0%)	<0.005
Both surgery and radiation	330 (0.5%)	2 (0.8%)	328 (0.5%)	<0.005
Actuarial survival by treatment (years ± SD)^*∗∗*^				
No treatment	1.253 ± 0.046	2.072 ± 0.598	1.243 ± 0.046	<0.005
Surgery only	8.560 ± 0.297	13.107 ± 1.306	8.324 ± 0.302	<0.001
Radiation only	1.190 ± 0.064	1.807 ± 0.447	1.189 ± 0.064	<0.001
Both surgery and radiation	3.649 ± 0.499	13.667 ± 13.500	3.287 ± 0.387	<0.005
Actuarial survival by morphology (years ± SD)^*∗∗*^				
Pleomorphic	1.591 ± 0.653	—	1.591 ± 0.653	—
Clear cell	2.526 ± 0.215	1.833 ± 1.090	2.526 ± 0.215	0.832
Spindle cell	0.628 ± 0.149	—	0.628 ± 0.149	—
Scirrhous	2.228 ± 0.362	—	2.228 ± 0.362	—
Fibrolamellar	6.905 ± 0.617	9.110 ± 1.204	6.016 ± 0.653	0.002
NOS	2.688 ± 0.072	6.529 ± 0.881	2.650 ± 0.072	<0.001
Overall mortality, *N* (%)				
Alive	11,538 (18.1%)	88 (34.2%)	11,450 (18.0%)	<0.001
Dead	52,233 (81.9%)	169 (65.8%)	52,064 (82.0%)	<0.001
Cancer specific mortality, *N* (%)				
Alive	11,538 (18.1%)	88 (34.2%)	11,450 (18.0%)	<0.001
Cancer death	39,999 (62.7%)	156 (60.7%)	39,843 (62.7%)	<0.001
Noncancer death	12,234 (19.2%)	13 (5.1%)	12,221 (19.3%)	<0.001
Cumulative survival (%)				
1-year		61%	34%	<0.001
2-year		47%	23%	<0.001
5-year		30%	12%	<0.001

*N* = number; SD = standard deviation; ^*∗∗*^data presented for patients with available information only.

**Table 4:** Demographic profiles of fibrolamellar and nonfibrolamellar hepatocellular carcinoma for 63,514 adults and 257 pediatric patients from the Surveillance, Epidemiology, and End Results (SEER) database, 1973–2011.

	Total		Pediatrics		Adults		*p* value
	fHCC	Non-fHCC	fHCC	Non-fHCC	fHCC	Non-fHCC	
*N* (%)	274 (0.4%)	63,497 (99.6%)	62 (24.1%)	195 (75.9%)	212 (0.3%)	63,302 (99.7%)	—
Age, (mean ± SD)	64 ± 12	63 ± 12	14 ± 4	13 ± 4	64 ± 12	63 ± 12	—
Gender, *N* (%)							
Male	164 (59.9%)	47,698 (75.1%)	34 (54.8%)	119 (61.0%)	130 (61.3%)	47,579 (75.2%)	<0.001
Female	110 (40.1%)	15,799 (24.9%)	28 (45.2%)	76 (39.0%)	82 (38.7%)	15,723 (24.8%)	<0.001
Race, *N* (%)^*∗∗*^							
Caucasian	175 (64.1%)	31,955 (50.5%)	35 (56.5%)	93 (48.4%)	140 (66.4%)	31,862 (50.5%)	NS
African American	32 (11.7%)	7,797 (12.3%)	9 (14.5%)	20 (10.4%)	23 (10.9%)	7,777 (12.3%)	NS
Hispanic	40 (14.7%)	10,245 (16.2%)	15 (24.2%)	34 (17.7%)	25 (11.8%)	10,211 (16.2%)	NS
Asian/Pacific Islander/Native Americans	26 (9.5%)	13,321 (21.0%)	3 (4.8%)	45 (23.4%)	23 (10.9%)	13,276 (21.0%)	NS

fHCC = fibrolamellar hepatocellular carcinoma; *N* = number; NS = not statistically significant with *p* value > 0.05; SD = standard deviation; ^*∗∗*^data presented for patients with available information only.

**Table 5:** Tumor characteristics of fibrolamellar and nonfibrolamellar hepatocellular carcinoma for 63,514 adults and 257 pediatric patients from the Surveillance, Epidemiology, and End Results (SEER) database, 1973–2011.

	Total		Pediatrics		Adults		*p* value
	fHCC	Non-fHCC	fHCC	Non-fHCC	fHCC	Non-fHCC	
*N* (%)	274 (0.4%)	63,497 (99.6%)	62 (24.1%)	195 (75.9%)	212 (0.3%)	63,302 (99.7%)	—
Grade, *N* (%)^*∗∗*^							
Well differentiated	22 (26.8%)	7,791 (35.1%)	6 (37.5%)	28 (35.4%)	16 (24.2%)	7,763 (35.1%)	NS
Moderately diff.	41 (50.0%)	8,426 (38.0%)	8 (50.0%)	25 (31.6%)	33 (50.0%)	8,401 (38.0%)	NS
Poorly diff.	19 (23.2%)	5,298 (23.9%)	2 (12.5%)	20 (25.3%)	17 (25.8%)	5,278 (23.9%)	NS
Undifferentiated	0 (0.0%)	672 (3.0%)	0 (0.0%)	6 (7.6%)	0 (0.0%)	666 (3.0%)	NS
Stage, *N* (%)^*∗∗*^							
Localized	96 (37.2%)	25,659 (48.0%)	15 (25.0%)	53 (29.1%)	81 (40.9%)	25,606 (48.1%)	<0.001
Regional	86 (33.3%)	16,691 (31.2%)	24 (40.0%)	70 (38.5%)	62 (31.3%)	16,621 (31.2%)	<0.001
Distant	76 (29.5%)	11,117 (20.8%)	21 (35.0%)	59 (32.4%)	55 (27.8%)	11,058 (20.8%)	<0.001
Size, *N* (%)^*∗∗*^							
Microscopic	0 (0.0%)	45 (0.1%)	0 (0.0%)	0 (0.0%)	0 (0.0%)	45 (0.1%)	NS
Under 2 cm	4 (2.0%)	3,067 (7.9%)	1 (1.8%)	15 (14.0%)	3 (2.0%)	3,052 (7.9%)	NS
2 to 4 cm	23 (11.2%)	11,697 (30.1%)	4 (7.3%)	13 (12.1%)	19 (12.7%)	11,684 (30.1%)	NS
Over 4 cm	178 (86.8%)	24,077 (61.9%)	50 (90.9%)	79 (73.8%)	128 (85.3%)	23,998 (61.9%)	NS

cm = centimeters; diff. = differentiated; fHCC = fibrolamellar hepatocellular carcinoma; *N* = number; NS = not statistically significant with *p* value > 0.05; NOS = not otherwise specified; SD = standard deviation; ^*∗∗*^data presented for patients with available information only.

**Table 6:** Treatment and survival outcomes of fibrolamellar and nonfibrolamellar hepatocellular carcinoma for 63,514 adults and 257 pediatric patients from the Surveillance, Epidemiology, and End Results (SEER) database, 1973–2011.

	Total		Pediatrics		Adults		*p* value
	fHCC	Non-fHCC	fHCC	Non-fHCC	fHCC	Non-fHCC	
*N* (%)	274 (0.4%)	63,497 (99.6%)	62 (24.1%)	195 (75.9%)	212 (0.3%)	63,302 (99.7%)	—
Treatment, *N* (%)^*∗∗*^							
No treatment	121 (45.1%)	45,437 (74.7%)	19 (30.6%)	99 (52.9%)	102 (49.5%)	45,338 (74.7%)	<0.001
Surgery only	133 (49.6%)	12,636 (20.8%)	41 (66.1%)	80 (42.8%)	92 (44.7%)	12,556 (20.7%)	<0.001
Radiation only	7 (2.6%)	2,452 (4.0%)	2 (3.2%)	6 (3.2%)	5 (2.4%)	2446 (4.0%)	<0.001
Both surgery and radiation	7 (2.6%)	323 (0.5%)	0 (0.0%)	2 (1.1%)	7 (3.4%)	321 (0.5%)	<0.001
Mean overall survival, (years ± SD)			9.110 ± 1.204	6.424 ± 0.866	6.016 ± 0653	2.657 ± 0.072	<0.001
Overall mortality, *N* (%)							
Alive	93 (33.9%)	11,445 (18.0%)	32 (51.6%)	56 (28.7%)	61 (28.8%)	11,389 (18.0%)	<0.001
Dead	181 (66.1%)	52,052 (82.0%)	30 (48.4%)	139 (71.3%)	151 (71.2%)	51,913 (82.0%)	<0.001
Cancer specific mortality, *N* (%)							
Alive	93 (33.9%)	11,445 (18.0%)	32 (51.6%)	56 (28.7%)	61 (28.8%)	11,389 (18.0%)	<0.001
Cancer death	154 (56.2%)	39,845 (62.8%)	30 (48.4%)	126 (64.6%)	124 (58.5%)	39,719 (62.7%)	<0.001
Noncancer death	27 (9.9%)	12,207 (19.2%)	0 (0.0%)	13 (7.7%)	27 (12.7%)	12,194 (19.3%)	<0.001

fHCC = fibrolamellar hepatocellular carcinoma; *N* = number; NS = not statistically significant with *p* value > 0.05; SD = standard deviation; ^*∗∗*^data presented for patients with available information only.

**Table 7:** Comparing survival outcomes of fibrolamellar and nonfibrolamellar hepatocellular carcinoma from the Surveillance, Epidemiology, and End Results (SEER) database, 1973–2011.

	fHCC	Non-fHCC	*p* value
*N* (%)	274 (0.4%)	63,497 (99.6%)	—
Survival by treatment, (years ± SD)^*∗∗*^			
No treatment	1.916 ± 0.464	1.246 ± 0.046	<0.001
Surgery only	11.325 ± 0.966	8.345 ± 0.296	<0.001
Radiation only	1.111 ± 0.348	1.190 ± 0.064	<0.001
Both surgery and radiation	7.393 ± 2.937	3.475 ± 0.489	<0.001
Cumulative survival (%)^*∗∗*^			
1-year	67%	34%	<0.001
2-year	50%	23%	<0.001
5-year	31%	12%	<0.001

fHCC = fibrolamellar hepatocellular carcinoma; *N* = number; SD = standard deviation; ^*∗∗*^data presented for patients with available information only.
